# Ultimate and proximate explanations of strong reciprocity

**DOI:** 10.1007/s40656-017-0151-4

**Published:** 2017-08-23

**Authors:** Jack Vromen

**Affiliations:** Faculty of Philosophy, Erasmus School of Economics, Erasmus University Rotterdam, P.O. Box 1738, 3000 DR Rotterdam, The Netherlands

**Keywords:** Strong reciprocity, Ultimate explanation, Proximate explanation, Reciprocal causation

## Abstract

Strong reciprocity (SR) has recently been subject to heated debate. In this debate, the “West camp” (West et al. in Evol Hum Behav 32(4):231–262, 2011), which is critical of the case for SR, and the “Laland camp” (Laland et al. in Science, 334(6062):1512–1516, 2011, Biol Philos 28(5):719–745, 2013), which is sympathetic to the case of SR, seem to take diametrically opposed positions. The West camp criticizes advocates of SR for conflating proximate and ultimate causation. SR is said to be a proximate mechanism that is put forward by its advocates as an ultimate explanation of human cooperation. The West camp thus accuses advocates of SR for not heeding Mayr’s original distinction between ultimate and proximate causation. The Laland camp praises advocates of SR for revising Mayr’s distinction. Advocates of SR are said to replace Mayr’s uni-directional view on the relation between ultimate and proximate causes by the bi-directional one of reciprocal causation. The paper argues that both the West camp and the Laland camp misrepresent what advocates of SR are up to. The West camp is right that SR is a proximate cause of human cooperation. But rather than putting forward SR as an ultimate explanation, as the West camp argues, advocates of SR believe that SR itself is in need of ultimate explanation. Advocates of SR tend to take gene-culture co-evolutionary theory as the correct meta-theoretical framework for advancing ultimate explanations of SR. Appearances notwithstanding, gene-culture coevolutionary theory does not imply Laland et al.’s notion of reciprocal causation. “Reciprocal causation” suggests that proximate and ultimate causes interact simultaneously, while advocates of SR assume that they interact sequentially. I end by arguing that the best way to understand the debate is by disambiguating Mayr’s ultimate-proximate distinction. I propose to reserve “ultimate” and “proximate” for different sorts of explanations, and to use other terms for distinguishing different kinds of causes and different parts of the total causal chain producing behavior.

## Introduction

Past attempts to bring insights and theories from evolutionary biology to bear on economics have taken various forms (cf. Vromen 2004). One form has been to argue not only that at a suitably general and abstract level, ongoing economic change exhibits the same principles as Darwinian evolution by natural selection, but also that economics can be improved by being explicitly based on those Darwinian principles. Examples of such an *analogical* connection include Generalized Darwinism (cf. Hodgson and Knudsen 2010) and applications of evolutionary game theory to analyze both the dynamic of learning processes (cf. Weibull 1995) and of the evolution of social norms (cf. Young 2015). An altogether different second form has been to argue that important determinants of current economic behavior such as (basic) preferences have been shaped in prior evolutionary processes. A case in point here is the Indirect Evolutionary Approach pioneered by Güth and Yaari (1992). It is important to note that arguing for the second *causal* connection is consistent with maintaining that ongoing economic change is not Darwinian in a meaningful sense (and this is indeed what proponents of the Indirect Evolutionary Approach argue). Then, finally, there is a third form of connecting biology to economics: arguing that zooming in on lower “biological” levels (or layers) of reality, such as the cellular level (think of neurons in the brain; see Glimcher 2010 on neuroeconomics) or the molecular level (think of genes), sheds an interesting light on current economic behavior. Again, pursuing this third, *ontological* connection need not commit one to believing that any of the other two connections is meaningful and worthwhile.

In previous work I stressed the independence of the three connections. Here I want to focus on a recent example in economics in which it is argued (or so I shall claim) that forging all three forms of connections is meaningful and worthwhile; an example which I will label The Case for Strong Reciprocity (henceforth abbreviated as SR). As a first approximation (to be refined below), one could say that SR refers to the predisposition of people to incur personal costs in punishing norm violators (this is sometimes called negative SR or altruistic punishment) and in rewarding norm adherents (sometimes called positive SR). Advocates of SR argue that SR is of great importance in producing and sustaining human cooperation. I call this The Case for SR because SR is contested. It is contested by some that SR exists at all, for example, and also by some that SR provides an acceptable ultimate explanation of human cooperation.^1^ In their Case for SR, advocates weave together the three forms of connections between evolutionary biology and economics mentioned above. First, advocates of SR often stress the significance of cultural evolution in fostering human cooperation. Thus advocates of SR hold that a meaningful analogical connection can and should be forged between ongoing cultural change and Darwinian biological evolution. Advocates of SR also hold that SR is a social preference that has been produced in prior gene-culture co-evolutionary processes. Thus they also forge the second, causal form of connection between evolutionary biology and economics. Finally, advocates of SR take recourse to neuroscience (and neuroeconomics), which study neural activity in the brain, to find out about the psychological machinery, and especially the motivations, underpinning SR. Thus also the third form of connection between biology and economics is pursued here.

In arguing all this, I maintain that in their Case for SR advocates of SR distinguish between ultimate and proximate explanation (Mayr 1961). In their gene-culture co-evolutionary models they aim at providing ultimate explanations of SR. That is, they develop new models in the spirit of gene-culture co-evolutionary theory in order to show how SR could have evolved under pressure of natural selection. And in their neuroeconomic studies they pursue proximate explanations of SR. That is, they engage in brain imaging in order to shed light on the specific sort of motivation underlying SR. In arguing this, I go both against Laland et al. (2014, 2011) who argue that the work of advocates of SR shows that Mayr’s ultimate—proximate distinction is no longer useful and against West et al. (2007, 2011) who argue that in their work advocates of SR mix up ultimate and proximate explanation in an unacceptable way. I argue, first, that Mayr’s ultimate—proximate distinction can be disambiguated in a way that is acceptable for both camps, that of Laland et al. and that of West et al., and, second, that in the work of advocates of SR the disambiguated distinction is not blurred. But before I come to this, let me first discuss in some detail what is involved in the case for SR and what Mayr’s ultimate—proximate distinction entails.

## The case for SR

Starting roughly at the last millennium turn, a collective of scholars, with Ernst Fehr, Robert Boyd, Herbert Gintis and Samuel Bowles as key figures (cf. Gintis et al. 2005), grouped together to make a case for SR. I call this the Case for SR not only because their work has met resistance from several quarters, but also because the scholars working on SR (whom I will henceforth call advocates) deliberately distance themselves from what they call standard biological and economic explanations of human cooperation. In particular, advocates of SR argue that standard biological explanations such as kin selection and reciprocal altruism, on the biological side, and standard economic theories that assume selfish motives for cooperation fall short of explaining distinctive features of human cooperation. There is a need for novel biological and economic theories, they argue (cf. Gintis et al. 2005), and those are what they set out to provide. Given that advocates of SR work on different disciplinary frontiers, getting a comprehensive overview of their work and of how the various parts relate to each other is not easy and straightforward. Indeed, as we shall see, the complexity of their project gave rise to several misunderstandings.

Let me start with some preliminary remarks about SR. Above I argued that, as a first approximation, SR refers to the predisposition of people to incur personal costs in punishing norm violators (this is sometimes called negative SR or altruistic punishment) and in rewarding norm adherents (sometimes called positive SR). A classic seminal study is Fehr and Gächter (2002). In a one-shot public goods experiment, Fehr and Gächter introduced an extra behavioral opportunity for subjects. Next to the standard options of contributing (“cooperating”) and non-contributing (“free riding”, or “cheating”) to the public good, subject were given the opportunity to incur personal costs in punishing non-contributors. Some 84% of the subjects actually seized this opportunity at least once. What is more, introducing this extra option proved to be an effective antidote against the standard decline of contributions over time (when the one-shot game is repeated several times) that is observed in standard public goods experiments that do not allow for costly punishments (Ledyard 1995). Instead of a gradual decrease of mean contributions without costly punishment, Fehr and Gächter observe a gradual increase of mean contributions with costly punishment.

For advocates of SR, this and other experimental work establishes not only that Strong Reciprocators actually exist (i.e., there are actually people out there in the real world who have, and act upon, SR as a social preference), but also that SR can be a potent force in sustaining cooperation. Fehr and Gächter call the costly punishment option that they introduce in their public goods experiment altruistic, because punishment does not yield material benefits for the punisher (Fehr and Gächter 2002, p. 137). For advocates of SR this is a crucial addendum to the definition of SR because it allegedly assures that SR cannot possibly be explained by the standard biological and economic theories alluded to above.^2^ If punishers would reap material benefits from their punishing behavior, then the biological theory of reciprocal altruism and the economic theory of cooperation that assumes selfish motives would have no problem in explaining the punishing behavior. Thus, in the writings of advocates of SR we typically find definitions of SR as the following: Strong Reciprocity is the social preference motivating people to sacrifice their own payoffs in order to cooperate with others, to reward the cooperation of others, and to punish free-riding, even when they cannot expect to gain from acting this way (Bowles and Gintis 2011, p. 20).

Advocates of SR do not rest content with showing that SR exists. They also want to explain why people have SR as one of their preferences. To this end they develop new evolutionary models with which they can show that SR could have evolved. What is crucially missing in standard biological accounts of human cooperation, they argue, is cultural evolution, and notably cultural group selection. And they also argue that what are crucially missing in standard economic models are social preferences such as SR: room should be made for SR as a separate argument in the utility function. Later on we will see that with respect to the latter advocates of SR want to dig deeper. They want to investigate what sort of motive underlies SR. It is important to note that advocates of SR are working on two fronts here. They want to enrich both standard biological theory and standard economic theory.

Let’s first discuss their evolutionary explanations of SR and in particular the role that culture and cultural evolution play in their evolutionary models. Here already we come across a misunderstanding of what advocates of SR supposedly argue, namely that in their view SR and the sort of human cooperation that SR produces would be the outcome of cultural evolution (and notably of cultural group selection) rather than biological evolution (cf. El Mouden et al. 2014). Although advocates of SR are adamant in stressing that cultural evolution played a key role in the evolution of SR, they do not hold that cultural evolution alone can explain SR.

Advocates of SR clearly believe not only that cultural evolution is a Darwinian process analogical to biological evolution, but also that cultural evolution and its products played a key role in the evolution of human cooperation. Let me elaborate on each point in turn. First, what does it mean to believe that cultural evolution is a Darwinian process, analogical to biological evolution, in its own right? Roughly, that key principles of Darwinian biological evolution, understood at a general and abstract level, also apply to culture and in particular to cultural change. Most of the time three such principles are identified: the principle of variation (that there is variation in traits), the principle of heredity (that offspring resemble their parents in relevant respects) and the principle of differential fitness (that individuals with different variants leave different numbers of offspring; Lewontin 1985, p. 7). The idea is that genetic inheritance is not the only way in which the principle of heredity can be satisfied. In cultural evolution, the social transmission of traits (as when an individual “socially” learns from other individuals—its “cultural parents”—what social norm to follow in a particular situation) fits the bill. This difference in mode of transmission (or replication)—genetic inheritance or social learning—is seen as the most important difference between biological and cultural evolution.^3^

It is important to see what is *not* implied by such a view. First, social learning need not be as faithful or as “high-fidelity” as genetic inheritance. Genetic inheritance, it is sometimes argued, is largely preservative. Copying and editing errors are relatively rare. By contrast, it is often argued that systematic biases in social learning (and thus also in cultural transmission) are the rule rather than the exception. Especially Dan Sperber and associates have been consistently arguing that a Darwinian perspective on culture based on the assumption that there are items in culture that are replicators (sometimes called memes; Dawkins 1976)—just like genes in biological evolution—is fundamentally mistaken (cf. Claidière et al. 2014). Rather than assuming that cultural transmission is a matter of faithful copying, Sperber urges that cultural transmission is systematically biased by what he calls attractors. Most of the time, when people try to learn something from others, they have to make inferences. The meanings that words have, for example, cannot be observed or imitated, but have to be inferentially reconstructed. In those processes of inferential reconstruction, pre-existing psychological biases, as “attractors”, play a key role.^4^

Sperber and his associates have used this argument repeatedly to resist and oppose attempts to Darwinize culture (Aunger 2000). But advocates of SR, such as Boyd, Richerson and Henrich, have consistently argued that Sperber’s argument, which they take to be well-taken for the most part, does not undermine their view that cultural change can be seen as a Darwinian evolutionary process in its own right. Just like Sperber, Boyd, Richerson and Henrich acknowledge the existence of a host of systematic biases in cultural transmission. Not only do people selectively choose their role models—the people they want to learn from—(these they call context biases), what they learn from them is also greatly affected by psychological mechanisms (what they call content biases). What is more, Henrich and Boyd (2002) argue that standard formal ways to model evolution by natural selection, such as the Price equation and replicator dynamic, can accommodate the systematic content biases stressed by Sperber and others. Henrich and Boyd show that the existence of strong Sperberean attractors might in fact speed up processes described by these formal modeling techniques.^5^

Thus, advocates of SR agree with Sperber and other critics that there are significant differences between cultural and biological evolution, yet maintain *pace* Sperber that it is worthwhile and fruitful to conceive of cultural change as a Darwinian process in its own right. But this is just one part of their story. Here Vromen’s (2004) distinction between the three clusters comes in handy. Conceiving of cultural change as a Darwinian process in its own right connects cultural change and Darwinian biological evolution analogically. Advocates of SR also connect cultural evolution and biological evolution causally, however. This is the other part of their story. With their gene-culture co-evolutionary theory, they want to bring out that that the causal connection between biological and cultural evolution is not a one-way street. It is not just that biological evolution and its products causally affect subsequent processes of cultural evolution. Conversely, cultural evolution and its products also causally affect subsequent processes of biological evolution. Sure, they argue, humans only could start cumulative cultural evolution after the requisite psychological capacities and mechanisms first evolved biologically. And biologically pre-evolved psychological mechanisms might still greatly affect ongoing processes of cultural evolution, as Sperber, evolutionary psychologists and advocates of gene-culture co-evolutionary theory alike recognize. But vice versa in the history of humankind cultural evolution and its products also determined to a large extent the social “environmental” pressures for subsequent processes of biological evolution. In a social world replete of culturally pre-evolved social norms, for example, in which people who fail to adhere to the prevailing norms are shunned from food-sharing and mating opportunities, the genetic reproductive success of people greatly depends on whether they are capable of internalizing the norms. In such a world, there is a tight pressure favoring the biological evolution of a “norm psychology”.^6^

Gene-culture co-evolutionary theory thus involves more than the recognition that culture has an inheritance mechanisms of its own. It also crucially involves the recognition that cultural evolution and its products change the conditions for subsequent biological evolution. In fact, since it transpired that cultural evolution also leaves *biological* traces, it would be more accurate to say that cultural evolution and its products change the conditions for subsequent *genetic* (not biological) evolution. The nascent subdiscipline of cultural neuroscience (cf. Chiao 2009; see also Henrich et al. 2010) shows, for example, that being raised in different cultures leads to different wirings of the brain. Enculturated brains differ from non-enculturated brains and the brains of enculturated people in the one culture differ from those of enculturated people in another culture. Thus culture turns out to have effects on biological organization at higher layers (or levels) than the molecular layer of the gene.^7^ In short, rather than maintaining that culture has its own realm or sphere, in ways similar to but also fully detached from the biological realm, advocates of SR, who also propagate gene-culture co-evolutionary theory, hold that the cultural and biological realm are deeply intertwined. Not only are there myriad ways in which processes of cultural evolution and processes of genetic evolution mutually affect each other, cultural evolution and its products also have direct biological consequences.

In their otherwise excellent analysis of cultural evolution and how it could produce prosocial behavior, El Mouden et al. (2014) erroneously assume that advocates of SR connect cultural and genetic evolution only analogically. El Mouden et al. (2014) discuss cultural evolution as if it were a parallel process to genetic evolution; a process analogous to genetic evolution, made possible by antecedent genetic evolution, but being unable to causally “feedback” on genetic evolution. They first rightly note that with cultural evolution not only does the principle of heredity get a different content, but also that the notions of fitness and natural selection get a different meaning. “Cultural fitness” refers to the overall influence that a cultural trait has on cultural traits in a descendant generation. Accordingly, “cultural natural selection” means that “descendants” select their cultural role models as those they want to learn from (ibid., p. 233). El Mouden et al. (2014) correctly observe that since cultural fitness and cultural natural selection might be altogether different from genetic fitness and genetic natural selection, the products of cultural evolution might also be altogether different from genetic evolution. As especially “memeticists” have emphasized, there can be runaway cultural evolution in which the “survivors” have low or even zero genetic fitness. Theories of cultural evolution might thus be able to explain “the Paris Hilton” effect (Henrich 2016, p. 126), the phenomenon that someone like Paris Hilton is becoming more and more famous for no other reason than that she is already quite famous.

Yet El Mouden et al. (2014) also rightly note that it is possible that cultural and genetic fitness correlate empirically. This might happen, for example, if social learners tend to pick out prestigious individuals as their cultural role models and if high prestige tends to be positively correlated with genetic reproductive success. If those two conditions are met, cultural and genetic evolution do not drift apart, but tend to result in similar, if not the same, outcomes. This example is interesting because advocates of SR who also advocate gene-culture co-evolutionary theory have always argued that the tendency of social learners to select prestigious individuals as their role models presents one of the most common context biases in social transmission. As El Mouden et al. suggest, this bias might have been shaped by prior processes of genetic evolution (ibid, p. 235). If so, prior processes of genetic evolution produced indirectly see to it, through producing systematic biases in cultural evolution, that cultural evolution does not deviate too much from the dictates of genetic evolution.

More in general, advocates of gene-culture co-evolutionary theory tend to stress that “Cultural evolution can solve many of the same adaptive problems as genetic evolution” (Henrich 2016, p. 239). Cultural evolution can do so by different means: via cultural transmission rather than genetic inheritance. Some evolutionary theorists have argued that natural selection is the only systematic force we know of that is capable of producing functional adaptedness (or the appearance of functional design). Perhaps. But if so, advocates of gene-culture co-evolutionary theory insist that natural selection should then be understood not too narrowly (as comprising only genetic natural selection), but broadly (as encompassing cultural natural selection).^8^

But what advocates of gene-culture co-evolutionary theory stress even more is that just as processes of cultural evolution draw on prior processes of genetic evolution and their products, processes of genetic evolution also draw on prior processes of cultural evolution and their products. Prior processes of cultural evolution and their products determine to a large degree the selection pressures for subsequent processes of genetic evolution. Indeed, someone as Henrich goes as far as stating that “… the *central force* driving human genetic evolution for hundreds of thousands of years, or longer, has been cultural evolution” (Henrich 2016, p. 316). This key aspect of gene-culture co-evolutionary theory is completely missed by El Mouden et al. (2014). El Mouden et al. (2014) do a great job in spelling out what is involved in seeing cultural change as a Darwinian process in its own right. They also render us a service in identifying the conditions under which cultural and genetic evolution tend to steer in the same direction. But they fail to do justice to what is arguably the most important hallmark of gene-culture co-evolutionary theory, namely that the causal connections between cultural and genetic evolution run both ways.^9^

Summing up now, advocates of SR put forward gene-culture co-evolutionary theory as the meta-theoretical framework that is capable of providing satisfactory explanations of the evolution of human cooperation and the role that SR plays in this. Although cultural evolution plays a key role in those explanations, most of the time genetic evolution also plays a role. Cultural evolution is seen as a Darwinian process in its own right that is analogous to genetic evolution. But advocates of SR do not claim that SR is the product solely of cultural evolution. Rather, the idea is that prior processes of cultural evolution created novel conditions that were conducive to the genetic evolution of SR.

## Are ultimate and proximate explanations mixed up in the case for SR?

West et al. (2011, p. 242) and Scott-Phillips et al. (2011, p. 41) (henceforth called the West camp) criticize the work of advocates of SR for conflating ultimate and proximate factors. The West camp argues, first, that SR is defined by its advocates as a proximate factor and, second, that advocates of SR put forward SR as a solution to the ultimate problem of why human cooperate. Since the West camp draws on Mayr (1961) in insisting that ultimate and proximate issues should not be mixed up like this, it believes this is a major drawback in the work on SR. In this section, I argue that the West camp is right in treating SR as a proximate factor, but wrong in asserting that advocates of SR put forward SR as an ultimate explanation of human cooperation. *Pace* the West camp, I argue that advocates of SR do not mix up ultimate and proximate explanation. Based on what I argued in the previous section, I argue that advocates of SR develop new models within the meta-theoretic framework of gene-culture co-evolutionary theory in order to provide ultimate explanations of human cooperation and the role played by SR in it. I furthermore argue that advocates of SR search for “deeper” proximate explanations of SR in neuroeconomic findings, based on the insight (also discussed in the previous section) that the causal interaction between cultural and genetic evolution leaves its traces in the human brain. But before I come to that, let me first spend a few preliminary words on Mayr’s (1961) classic distinction between ultimate and proximate causes.

Mayr (1961) distinguishes ultimate from proximate causes of animal behavior. Ultimate causes are invoked in answering the question Why—to serve what function—the behavior evolved in the first place. Proximate causes are invoked in answering How—through what causal interaction between the animal’s behavioral machinery and environmental factors—the behavior is produced. For example, Mayr discusses bird migration as follows. A plausible ultimate explanation is that ancestors of the present population of migrating birds have been reproductively more successful than birds that did not migrate. In short, there has been natural selection for migration. A plausible proximate explanation is that a drop in temperature or a shortening of daylight triggers behavioral mechanisms in members of the present population of migrating birds, which in turn make them migrate. Mayr stresses that both sorts of explanations are legitimate and worthwhile, that we need both for a complete understanding of animal behavior and that the two sorts of explanations are complements rather than rivals. Below we will see that despite its apparent clarity, several aspects are packed into Mayr’s ultimate—proximate distinction that should be disambiguated. But for now it suffices to note that Mayr warns against mixing up the two sorts of explanations: typically, an ultimate cause should not be invoked in a proximate explanation and, conversely, a proximate cause should not be invoked in an ultimate explanation.

Some further preliminary remarks and caveats are in order. I do not want to deny that what advocates of SR argue is at times confusing. Not all advocates of SR speak in the same voice and not even each advocate’s assertions are consistent over time. I think this is so for several reasons. Of course, one obvious reason might be that advocates changed their mind. But another reason is surely also that the advocates have different backgrounds and have different battles to fight. Central notions such as “altruism” have different meanings in different audiences (Clavien and Chapuisat 2013; Kitcher 2010). What is also confusing is that advocates of SR have put forward many different ultimate explanations of SR. Some of them invoke group selection, others don’t.

Experimentalists such as Fehr, Fischbacher and Gächter sometimes seem to suggest that their experimental findings shed light both on ultimate and proximate explanations of SR.^10^ They sometimes suggest that the sort of motivations strong reciprocators are led by can be inferred directly from the findings. And they suggest that the (alleged) fact that the presence of strong reciprocators helps to sustain high levels of cooperation partly explains how SR could have evolved (cf. Egas and Riedl 2008).^11^ But more often than not advocates of SR acknowledge that other theoretical and empirical work than experimental work is needed to provide ultimate and proximate explanations of human cooperation and the role played in it by SR.

Advocates of SR have put a lot of effort in contrasting Strong with Weak Reciprocity. They have contrasted SR in particular with Reciprocal Altruism (RA; Trivers 1971), which they see as one of Weak Reciprocity’s instantiations. RA is often discussed by advocates of SR as implying (or even being) a particular proximate mechanism: reciprocal altruists are said to be selfish in the sense that they only engage in costly helping acts because (and when) they expect to reap compensating benefits from the beneficiaries in the future. In fact this is completely mistaken. RA is an ultimate, not a proximate explanation of cooperation. As an ultimate explanation, RA is compatible with a host of proximate mechanisms. As Trivers (1971) himself carefully explains, shrewd calculation might underlie the conditional pattern of behavior described by RA, but also emotions such as generosity, gratitude and moral aggression. It is not strange for evolutionary biologists, who know that RA is not a proximate but an ultimate explanation of cooperation, to conclude that SR also is an ultimate explanation when they see that SR is contrasted by its advocates with RA.

That advocates of SR argue as if RA were (or implied) a particular proximate mechanism is strange for at least two reasons. One is that they acknowledge that ultimate explanations of cooperation “… remain silent as to the proximate motives leading humans to engage in helping, and sometimes altruistic, behaviors” (Bowles and Gintis 2011, p. 76). But if this is so, why do they ascribe a particular self-regarding motive to reciprocal altruists? Another reason why this is strange is that it has not escaped their attention that Trivers himself puts forward moralistic aggression as a plausible proximate mechanism that is quite different than shrewd selfish calculation. Advocates of SR even go as far as to suggest that moralistic aggression might underlie SR. As they contrast SR with RA especially at this point of different underlying motives this is very strange indeed!

Thus I do not argue that advocates of SR are always careful and consistent especially when it comes to distinguishing ultimate from proximate explanation. Yet I do argue in the rest of this paper that in their constructive work on SR they pursue ultimate and proximate explanation as two different explanatory enterprises that are fairly independent of each other.

We saw that advocates of SR define SR as a propensity or (pre)disposition to act in certain ways. The West camp is right in arguing that SR should be seen as a proximate, rather than ultimate cause of human cooperation. But what makes the West camp believe that advocates of SR put forward SR as an ultimate explanation of human cooperation? Apparently because the West camp sees the following passage as support for their claim: “Why do people punish violators of widely approved norms although they reap no offsetting material benefits themselves? We hypothesize that individuals derive satisfaction from the punishment of norm violators.” (De Quervain et al. 2004, p. 1254). The West camp apparently treats the first Why question here as a request for an ultimate explanation of human cooperation. If this were true, the West camp would definitely have been right in arguing that De Quervain’s explanatory hypothesis, which clearly refers to a proximate cause, does not fill the bill.

But the West camp is clearly wrong in assuming that the Why question posed in the first sentence is meant to be a request for an ultimate explanation. A careful reading of De Quervain’s paper makes it abundantly clear that they are looking for a plausible proximate mechanism underlying altruistic punishment (as the negative side of SR; ibid., p. 1254). De Quervain et al. are not providing an evolutionary model of altruistic punishment. Such evolutionary models, they argue, have already been provided in other work on SR. Here they refer to Boyd et al. (2003) and Bowles and Gintis (2004). Rather, they argue, their work is complementary to these. De Quervain et al. turn to neuroeconomics (and to results from PET-scans in particular) to find out about what specific motivation underlies altruistic punishment.^12^ They conclude that “Our results therefore support recently developed social preference models (6–8), which assume that people have a preference for punishing norm violations, and illuminate the proximate mechanism behind evolutionary models of altruistic punishment.” (ibid., p. 1258). Thus De Quervain et al. (2004) do distinguish between ultimate and proximate explanation, they want to do justice to it and they do so in a way that should not upset enthusiasts about the distinction.

Contrary to what the West camp apparently believes, not all Why questions call for an ultimate explanation. If one wants to find out about motivations underlying some behavior, as De Quervain et al. clearly want to do, it is quite normal to put this in a form of a Why question. One is reminded here of the example of the (in)famous bank robber Willie Sutton, discussed by Garfinkel (1981). Why did you rob banks, the reporter asks Willie Sutton. Because that is where the money is, answers Willy. The reporter might have been looking for another sort of proximate cause (not why Willie robbed banks rather than, say, gas stations, but why Willie did not choose to earn his money in a decent job), but it is clearly a proximate rather than an ultimate explanation that he is looking for.

Conversely, the ultimate Why question can be, and in fact often is, paraphrased in terms of a How question: how could some behavior or trait have evolved under pressure of natural selection.^13^ As natural selection is often taken to be the ultimate force at work, the idea is that an ultimate explanation points at the evolutionary problem that is solved by the behavior or trait. Or, to put it differently, an ultimate explanation should identify the function or purpose of the behavior and trait. The challenge is to put forward a plausible story, model or scenario in which it is shown that the behavior or trait at issue conferred superior lifetime or inclusive fitness to the individuals displaying the behavior (or the individuals having the trait). This challenge is especially daunting when it comes to providing ultimate explanations of altruistic and cooperative behavior, since the individuals displaying these behaviors incur fitness losses compared to cheaters.

What might be confusing here is that De Quervain et al. (2004) are looking for a proximate explanation of altruistic punishment, while altruistic punishment itself is seen as a proximate cause of human cooperation. Why would SR, as a proximate cause, be in need for a further (or “deeper”) proximate explanation? The reason, I submit, is that SR is a proximate cause of a rather shallow type. SR is understood as particular sort of propensity, (pre)disposition and social preference, we saw. What is says is that under normal circumstances, Strong Reciprocators incur personal costs in punishing norm violators. Only under extraordinary circumstances, if for example personal costs are too (or “prohibitively”) high, strong reciprocators might refrain from acting on their propensity.^14^ In definitions of SR it is typically not specified when strong reciprocators do act and when they do not act on their propensity. What is more, as Bowles and Gintis (2011, p. 9) make clear, a particular “revealed” preference that is ascribed to some agent on the basis of her behavior (cf. Savage 1954) tells next to nothing about the underlying psychological process leading to the behavior.^15^ In particular, it is silent about underlying motivations. In the case of SR, one might be tempted to assume that the underlying motivations are other-regarding. But in fact they could also be self-regarding (Bowles and Gintis 2011, p. 11). That is to say, it might be that strong reciprocators punish norm violators, not because they see this as their moral duty or because they want the violators to adhere to the norms, but ultimately because they expect psychic satisfaction from doing so. Indeed, as we saw before, De Quervain et al. (2004) conclude from their PET scan findings that altruistic punishers are psychologically selfish precisely in this sense.

I conclude that that the West camp is wrong in arguing that advocates of SR put forward SR as an ultimate explanation of human cooperation. Advocates of SR consistently treat SR as a proximate cause of human cooperation and they agree with the West camp that as such SR does not provide an ultimate explanation of human cooperation. Advocates of SR do put forward a particular sort of motivation as an answer to a Why question, but the Why question at issue is clearly meant to be a proximate and not an ultimate question. They do this because they believe SR is a rather shallow proximate cause in need of a deeper proximate explanation.

## Do advocates of SR hold that Mayr’s ultimate: Proximate distinction is no longer useful?

Partly based on the work of advocates of SR, Laland et al. (2013; 2011) call into question whether Mayr’s distinction is still useful. The Laland camp, which is clearly sympathetic to the work of SR’s advocates, argues that the time is ripe for a drastic revision, if not rejection, of Mayr’s distinction.^16^ In particular, it argues that the work of advocates of SR calls for a replacement of Mayr’s notion of uni-directional causation by a notion of reciprocal causation. I argue instead that there is a greater need for a disambiguation of Mayr’s distinction.

In the previous section I argued, *pace* the West camp, that SR, which all agree is a proximate cause, does not figure in ultimate explanations provided by advocates of SR. Rather gene-culture co-evolutionary theory is invoked to pursue ultimate explanations of SR. But this does not mean that proximate causes do not figure in ultimate explanations of human cooperation provided by advocates of SR. Psychological mechanisms driving cultural evolution do feature prominently as proximate causes in their ultimate explanations of human cooperation, namely as factors that significantly have changed the conditions for genetic evolution in the distant past.^17^

Viewing cultural evolution and its products in this way, as factors that significantly have changed the environment for subsequent genetic evolution, effectively brings gene-culture co-evolutionary models under the rubric of *niche construction theory* (Odling-Smee et al. 2003). Niche construction theory stresses that often organisms are not passively undergoing environmental pressure, but are actively altering and modifying their environments. It is first and foremost this insight that the Laland camp wants to capture with its notion of reciprocal causation. Rather than mixing up ultimate and proximate explanation as the West camp wrongly accuse the work on SR of, the Laland camp argues that the work of SR’s advocates points out the need for a new and richer notion of causation than the simple notion Mayr’s distinction allows for. The Laland camp argues forcefully that with his notion of unidirectional causation, in which the arrow of causation runs in one direction only, namely from environment to traits, Mayr presented a relatively rare phenomenon. The example of bird migration that Mayr gave to illustrate the difference between proximate and ultimate causes, was well-chosen to fit his notion of unidirectional causation (see also Sterelny 2013). But there are many more examples in nature, the Laland camp ventures, in which the arrow of causation runs both ways, from environment to traits and from traits to environment. The work of advocates of SR on the evolution of human cooperation is mentioned in Laland et al. (2011) as one of the four areas of recent research that prompt the supersession of Mayr’s notion of unidirectional causation by the notion of reciprocal causation.

At first sight, the argument put forward by the Laland camp might seem unassailable. If indeed proximate causes played a crucial role in shaping the selection pressures for genetic evolution, then it seems reference to these proximate causes in ultimate explanation is not only permissible but perhaps even mandatory. Mayr’s notion of unidirectional causation should be superseded by that of reciprocal causation, it seems, and his ban on invoking proximate causes in ultimate explanations should be lifted. But drawing these conclusions might be too quick and too easy.

First, as Dickins and Barton (2013) argue, the notion of reciprocal causation might be a bit misleading. For it suggests that the organisms’ traits and the environment mutually affect each other simultaneously. But in fact advocates of SR argue that the two causally affect each other sequentially, one after the other: genetic evolution gave rise to the proximate capacities necessary for cumulative cultural evolution, which in turn led to changed environmental conditions for genetic evolution. The notion of unidirectional causation might still be apt to account for this sequence of causal processes. It is just that the sequence might be more complex than Mayr (1961) envisaged.

Furthermore, Mayr’s distinction between proximate and ultimate causes as such does not rule out that the sort of causal scenario envisaged by niche construction theorists can be recognized. In fact, it can be argued (as Dickins and Barton 2013 do) that the causal scenario envisaged by niche construction theorists presupposes rather than obviates the proximate—ultimate distinction. After all, stressing that the proximate causes working in cultural evolution shaped the conditions for genetic evolution, as advocates of SR do, assumes that a meaningful distinction between proximate and ultimate causes can be made. Dickins and Barton and other members of the West camp also rightly argue that even if cultural evolution has been as important for the genetic evolution of social preferences as advocates of SR claim, this does not establish that the proximate causes involved in cultural evolution should be referred to in ultimate explanations of SR. In an acceptable ultimate explanation of SR, one could choose to start with just mentioning the conditions allegedly produced by earlier cultural evolution. That is, one could chose not to go further back in time in one’s explanation and not to look at the factors and processes that produced the conditions.

What the latter observations also show is that there need not be any factual disagreement between the Laland camp and the West camp. Both camps might agree about the causal processes involved in the evolution of social preferences such as SR (Laland et al. 2014). The disagreement between the two camps might be limited to how we should theoretically account for these causal processes. That is, the Laland camp holds that since cultural evolution and its products made such a big difference to what evolved genetically afterwards, an acceptable ultimate explanation should explicitly mention cultural evolution and the proximate mechanisms at work in it. Even though the West camp might grant the importance of cultural evolution and the proximate mechanisms at work in it in shaping the conditions for genetic evolution, it sees no need to adjust its belief that ultimate explanations should not invoke proximate mechanisms. The West camp can maintain this belief by arguing that ultimate explanations need not go so far back in time that the factors that produced the conditions for genetic evolution should also be taken into account in acceptable ultimate explanations.

As the West camp, the “skeptics” with respect to niche construction theory (Scott-Phillips et al. 2014), makes clear, evolutionary processes can and should be distinguished from the causes of evolutionary processes. Four evolutionary processes are traditionally recognized in the standard evolutionary synthesis, the West camp argues: natural selection (including sexual selection), genetic drift, mutation and migration. All four evolutionary processes lead to changes in gene frequencies. The West camp is adamant in stressing that ultimate explanation is inextricably tied to genetic evolution.^18^ But the West camp also emphasizes that this does not imply that non-genetic factors are ruled out as important causes. It has been long appreciated that many different causes might change conditions for genetic evolution, West et al. argue. Niche construction is just one of those, one that alters the local ecology. Thus the standard evolutionary synthesis can accommodate these causes, the West camp argues, without pre-judging which are more important or more crucial: evolutionary processes or the causes affecting them.

Without denying that this is a defensible take on niche construction, I think two observations are in order. One is that even though the West camp argues that the standard evolutionary synthesis does not prejudge the issue of whether evolutionary processes or causes are causally more powerful or efficacious, there might be a tendency to regard the former as basic and the latter as mere add-ons (cf. Laland et al. 2014, p. 164). In this vein, the basic evolutionary processes are “foregrounded” and the causes of evolutionary processes are “backgrounded”. If advocates of gene-culture co-evolutionary theory are right that cultural evolution has been the central force driving human genetic evolution (as Henrich 2016 argues) then a case can be made for reversing this way of picturing human evolution: cultural evolution, and the proximate causes involved in them, should be foregrounded and the basic evolutionary processes should be backgrounded.

This brings me to my second observation. Even if we accept the West camp view that ultimate explanation should be confined to basic evolutionary processes producing changes in gene frequencies, this does not establish that reference to proximate causes in ultimate explanation is not admissible. The West camp is right that ultimate explanation need not go into the causes of basic evolutionary processes. But this does not imply that it would be wrong or mistaken to push ultimate explanation further back in time. On the contrary: if the proximate mechanisms involved in cultural evolution played a crucial causal role in molding the conditions for genetic evolution, as advocates of gene-culture co-evolutionary theory hold, then this provides an excellent reason to explicitly refer to these proximate mechanisms in ultimate explanations.

## Disambiguating Mayr’s ultimate: proximate distinction

Laland et al. (2011) suggest that work on SR shows that Mayr’s ultimate—proximate distinction is no longer useful. In the previous section I argued that this suggestion is misleading. Laland et al. might be right that the work of advocates of SR calls for a richer “reciprocal” notion of causation, which allows for causal feedback from the organisms’ proximate mechanisms to the environment, than Mayr’s original unidirectional notion. But rather than superseding Mayr’s distinction between ultimate and proximate causes, the notion of reciprocal causation presupposes this distinction. What is more, as the West camp is eager to point out, in niche construction theory and gene-culture co-evolutionary theory the causal interaction between the organisms’ proximate mechanisms and the environment is not simultaneous but sequential. This entails that the standard view that proximate mechanisms should have no place in ultimate explanation can be maintained: proximate mechanisms can be seen as causes of evolutionary processes (shaping the conditions for these evolutionary processes) that need not (and the West camp insists, should not) be referred to in ultimate explanation.

What is perhaps most important for my present purposes, however, is that the West and Laland camps agree that it still is worthwhile not to mix up ultimate and proximate explanation. Despite all their disagreements, both camps agree that for each behavioral trait there are two explanatory projects worth pursuing. Appearances notwithstanding, the Laland camp does not plead for superseding Mayr’s ultimate—proximate distinction *in this sense*. They only plead for superseding Mayr’s ultimate—proximate distinction in another sense, namely that proximate mechanisms should not be referred to in ultimate explanation. If the proximate mechanisms at work in cultural evolution have been as important in the genetic evolution of SR as advocates of SR claim, the Laland camp argues, then these proximate mechanisms should be highlighted in ultimate explanation.

At first sight, Mayr’s ultimate—proximate distinction seems to be neat and clear-cut. But on closer inspection it turns out to be ambiguous and in need of disambiguation. Recall that Mayr introduces the distinction to mark different *kinds of causes*. Whereas for Mayr ultimate causes comprise the basic evolutionary processes mentioned by Scott-Phillips et al. (2014), and notably natural selection, proximate causes refer to the causal interaction between the DNA code of organisms and environmental conditions (or stimuli, as Mayr called them) immediately before, and leading to, the behavior at issue. Implicitly involved here is also a *temporal dimension*: ultimate causes precede proximate causes in time. And some would be inclined to say that ultimate causes produce particular frequencies of DNA codes in populations, as parts of proximate causes. But then there is also a distinction that Mayr draws between different *sorts of questions* that are asked and answered in different branches of biology. There is the How question asked in functional biology, which Mayr further specifies as: How do organisms respond to immediate environmental conditions in producing the behavior at issue?^19^ And there is the Why question asked in evolutionary biology, which Mayr further specifies as how come that the individuals of a species are endowed with their particular DNA code?

In short, there are at least three dimensions or aspects to Mayr’s ultimate—proximate distinction^20^:

1. A distinction between different kinds of causes: evolutionary processes (such as, notably, natural selection) and proximate mechanisms (such as the genetic make-up of organisms, but also possibly psychological or neurological mechanisms)—I propose to omit the phrases ultimate and proximate altogether here and call these *evolutionary processes* and *behavior*-*generating mechanisms* inside organisms respectively;
2. A distinction between different episodes in the total causal chain leading to the behavior: temporarily remote parts (“ultimate”) and temporarily nearby parts (“proximate”)—I propose to omit the phrases ultimate and proximate here too and call these *remote parts* and *nearby parts* respectively;
3. A distinction between different sorts of explanatory projects: ultimate explanations (typically answering the question: Why—to serve what function—did the behavior evolve?) and proximate explanations (typically answering the question: How is the behavior produced—what causal interaction between internal behavior-generating mechanisms and external conditions produced the behavior?)—I propose the reserve “ultimate” and “proximate” for these two sorts of explanations.

This disambiguation helps in identifying what is new in the ultimate explanations of SR put forward by SR’s advocates. And it helps in sorting out what the West and Laland camp agree about and what they disagree about.

All three, advocates of SR, the West camp and the Laland camp, agree that we ought to retain the distinction between ultimate and proximate explanation. They all agree that for a comprehensive understanding of some trait, such as SR, we need to pursue both ultimate and proximate explanation. They also agree about what should be involved in a satisfactory proximate explanation: a satisfactory proximate explanation should focus on the nearby parts of the causal chain producing the trait and more specifically on the causal interaction between behavior-generating mechanisms inside organisms and prevailing environmental conditions.

Disagreements between the three do not pertain to proximate explanation and what is involved in it, I submit, but only to what should and should not be involved in ultimate explanation. The West camp entertains a rather conventional and strict view on this. Ultimate explanations should invoke only evolutionary processes, the West camp insists, and should not refer to very remote parts in the causal chain leading up to the trait, or at least not to remote parts in which other causal factors than evolutionary processes played a crucial role. Let me elaborate a bit on both aspects. For the West camp the evolutionary processes that should be invoked in ultimate explanation are exclusively tied to genetic evolution. On the West camp’s view, explanations that invoke cultural natural selection, rather than genetic natural selection, and that do not explain changes in gene frequencies cannot possibly qualify as acceptable ultimate explanations. This is in marked contrast to the Laland camp, which in principle would accept explanations that invoke cultural natural selection and that do not explain changes in gene frequencies as *bona fide* ultimate explanation. As Mesoudi et al. (2013) and Henrich (2016) emphasize, since cultural evolution can be seen as a parallel process analogous to genetic evolution that is also capable of producing functional design-without-a designer, theories and models of cultural evolution can provide ultimate explanation just as theories and models of genetic evolution can. In other words, the Laland camp pleads for a broadening of the notion of ultimate explanation so that it can also encompass cultural evolution.

The Laland camp is also less strict about ultimate explanation than the West camp with respect to the temporal dimension. The Laland camp argues that there are good reasons to push the time horizon in ultimate explanation further back in time than the West camp is willing to do. Here gene-culture co-evolutionary theory, and especially its insight that cultural evolution and its products can, and allegedly actually did, greatly affect the conditions for subsequent genetic evolution, enters the picture. The Laland camp forcefully argues that if it is indeed true, as it surmises, that cultural evolution and its products have been a, if not the central force determining the conditions for human genetic evolution, then cultural evolution should be foregrounded rather than backgrounded in ultimate explanation. If it is furthermore true that psychological mechanisms (such as context and content biases in social learning) play a key causal role in cultural evolution, then the ban on invoking “proximate mechanisms” (what I propose to call behavior-generating mechanisms inside organisms) imposed by the West camp should be broken. In this paper I argued that this is a valid argument. The West camp might have a point that even if gene-culture co-evolutionary theorists are factually right in stressing behavior-generating mechanisms as causes of evolutionary processes, we can still choose not to take these remote parts of the causal chain (pertaining to these causes) into account in our ultimate explanations. But by thus backgrounding causes of evolutionary processes, the false impression is raised that the foregrounded standard evolutionary processes are doing the bulk of the explanatory work in human genetic evolution.

How does the work on SR done by SR’s advocates fit in all of this? Above I argued that most of the evolutionary models advocates of SR construct to give ultimate explanations of SR are within the confines of gene-culture co-evolutionary theory. To be more precise, in several of these models it is assumed that “leveling” institutions and social norms pre-evolved culturally that made conditions for the genetic evolution of SR more favorable because they attenuate within-group variation. As Bowles and Gintis note, this makes such models instances of niche construction theory (Bowles and Gintis 2011, p. 112). The task that advocates of SR set themselves in their pursuit of an acceptable ultimate explanation of SR is fairly standard and conventional: they think they have to show not only that genetic natural selection allows strong reciprocators to spread in populations and to sustain a sizable frequency in populations once they have spread, but also that genetic natural selection allows strong reciprocators to invade a population without strong reciprocators (ibid., p. 149). All this is perfectly in line with what the West camp believes should be established in ultimate explanation. The only point where the two diverge relates to where, in what temporal part of the total causal chain, ultimate explanation should start. Whereas the West camp holds that ultimate explanation should start with the niche constructed by cultural evolution (without mentioning the factors that go into the construction), advocates of SR agree with Laland et al. that, given that cultural evolution and its products are believed to be the crucial difference makers, ultimate explanations should start with the construction of the niche and with the causal factors that go into this.

This is a far cry from West et al.’s critique that SR, which is indeed defined as a proximate mechanism, is presented by its advocates as an ultimate explanation of human cooperation. What advocates of SR do in their ultimate explanations of SR is close to what the West camp believes should be done in ultimate explanations. This is not to say that the ultimate explanations of SR offered by its advocates might not have other shortcomings and possibly also deficiencies. It has been questioned by several commentators of Guala (2012), for example, whether the type of uncoordinated punishment implied by SR could have evolved in an ancient evolutionary environment without centralized authority. Furthermore, it has been contested (Lehmann and Keller 2006) that cultural evolution indeed constructs niches that are favorable to the genetic evolution of SR (cf. Molleman et al. 2013). The same holds for the proximate explanations of SR offered by its advocates. The use of the findings of neuro-imaging and of brain scans in identifying underlying motivations is also contested, for example. In his (in)famous critique of the reverse inference fallacy, Poldrack (2006) points at De Quervain et al. (2004) as one of the examples in neuroeconomics committing the fallacy. Thus there might be many things wrong with the specific evolutionary and proximate explanations of SR put forward by its advocates. But advocates do not go awry with mixing up ultimate and proximate explanation, let alone with proposing SR as an ultimate explanation of human cooperation.

## Conclusion

Although the three ways to bring evolutionary biology to bear on economics—analogical, causal and ontological—are often disconnected, I have argued that they are connected in the case for SR. In the case for SR, cultural change is believed to be analogous to genetic evolution, it is believed that gene-culture co-evolutionary theory points out how cultural evolution and genetic evolution interact causally in various ways and it is believed that findings from brain scans can shed light on the motivations underlying behavior. Furthermore, I have argued that for advocates of SR gene-culture co-evolutionary theory is the meta-theoretical framework for providing ultimate explanations of SR and that neuroeconomics is invoked to provide proximate explanations of SR. This view I have contrasted with the view voiced in the West camp, namely that SR is a proximate mechanism that is put forward by its advocates to provide an ultimate explanation of human cooperation. To be sure, SR is a proximate mechanism, but it is a rather shallow one in need for further proximate mechanism. And instead of SR itself being advanced as an ultimate explanation, advocates advance ultimate explanations of how SR could have evolved. I also argued that the notion of reciprocal causation that the Laland camp proposes fails to do justice to the work of SR’s advocates. It suggests that primate and ultimate causes causally interact simultaneously, while advocates of SR argue that they causally interact sequentially. What advocates of SR stress in particular, is that antecedent processes of cultural evolution constructed the niches for subsequent processes of genetic evolution. I argued that the best way to capture this is by disambiguating Mayr’s ultimate-proximate distinction. I proposed to reserve “ultimate” and “proximate” for different sorts of explanations, and to use other terms for distinguishing different kinds of causes and different parts of the total causal chain producing behavior. It was finally pointed out that this disambiguated notion of the ultimate—proximate distinction can be used not only for aptly characterizing what advocates of SR are doing, but also for specifying points of agreement and of disagreement between the West camp and the Laland camp.

## Footnotes

1 For a critical discussion of what exactly the laboratory experiments conducted by advocates of SR establish, see for example Guala (2012). For the critical argument that if the advocates of SR are right with their ultimate explanation, then the behavior they measure in their laboratory experiments must be maladaptive (contrary to what advocates of SR argue), see Johnson et al. (2003) and Burnham and Johnson (2005).

2 “Allegedly” because it is not clear that standard biological and economic theories really cannot explain what Fehr and Gächter (2002) call altruistic punishment. I won’t go into this here.

3 In addition, fitness might have to be redefined (as cultural fitness, meaning the expected success of individuals to serve as cultural role models that others learn from) and there might also be a reason to reinterpret (natural) selection (sometimes, cultural selection or cultural natural selection is spoken of).

4 Sperber (1996) has argued that attractors in cultural transmission need not be due to psychological factors. Attractors might also be caused by ecological factors. In what follows, however, I will assume for convenience that attractors are caused by psychological factors.

5 See Acerbi and Mesoudi (2015) for the argument that especially in the joint recognition of content biases in cultural transmission there is much more common ground between Sperber’s epidemiological, cultural attraction approach and gene-culture co-evolutionary theory than Sperber grants. Morin (2016) resists this argument.

6 As Henrich (2016, p. 189) explains, “norm psychology” entails both that humans intuitively assume that the social world is rule governed and that we tend to internalize norms (i.e., tend to treat them as goals in themselves) when we learn them. An interesting question—one that I cannot go into now—is whether something of a norm psychology is needed to get the cultural evolution of social norms off the ground.

7 Note that we enter Vromen’s third cluster of issues here (2004).

8 See Mesoudi et al. (2013) for a similar argument that models of cultural evolution can provide ultimate explanations of behavioral traits.

9 It is perhaps telling that El Mouden et al. (2014) speak of ‘dual-inheritance’ theory (p. 231), instead of gene-culture co-evolutionary theory. Although not wrong as a label, ‘dual-inheritance’ points at a different aspect (namely that there are two transmission mechanisms working side by side) than ‘co-evolutionary’ (namely that that there are mutual causal connections between them).

10 This has been criticized by the “Johnson camp” (or “Burnham–Johnson–Trivers camp”; cf. Johnson et al. 2003; Burnham and Johnson 2005; Trivers 2004): the Johnson camp holds that our present psychological dispositions very likely evolved in an ancient evolutionary environment in which repeated pairwise interactions rather than one-shot encounters between strangers were prevalent. For responses to this critique by advocates of SR, see for example Fehr and Gächter (2002) and Fehr and Henrich (2003).

11 The reason why is easy to fathom: groups with more cooperation outperform groups with less cooperation. Thus, if there is group selection strong enough to counter individual selection, then SR could have evolved. Although there is much more to be said about (cultural) group selection, I will not do that in this paper. See Vromen (2012) for some discussion of this.

12 For a recent example in the same spirit, see Hein et al. (2016).

13 Mayr (1961) already suggested that the ultimate Why question should be interpreted as a “How come” question.

14 Typically, it is not spelled out how sensitive the behavior responds to personal costs.

15 Thus, I basically agree with Rand and Nowak (2013), who similarly argue that Strong Reciprocity is a particular theoretical interpretation of behavior (what could be called a phenomenon, following Bogen and Woodward 1988) in need of an evolutionary explanation (and, I would like to add, also in need of a proximate explanation).

16 Note that the position taken by the Laland camp seems to be diametrically opposed to that of the West camp, which is critical of work on SR but sympathetic to retaining Mayr’s distinction.

17 In Marchionni and Vromen (2009), these are called then-working proximate causes (to contrast them with the “now”-working proximate causes that are invoked in proximate explanations of current behavior).

18 Note that this implies that a cultural evolutionary explanation of the fact that some trait is widespread cannot possibly be called an ultimate explanation. See Mesoudi et al. (2013) for a rebuttal of this view.

19 The generality of this question suggests that there might be other factors in organisms than their DNA codes, such as psychological mechanisms, that might explain how and why organisms respond to immediate environmental conditions the way they do.

20 There might be even more dimensions. One could argue, for example, that ultimate explanations typically are and proximate explanations typically are not population-level explanations. An anonymous referee pointed out that the first distinction seems to follow from the more fundamental third one: it is reference to evolutionary processes that answers ultimate Why questions and it is reference to behavior-generating mechanisms that answers proximate How questions. While I agree that it is hard to see what else could answer proximate How questions than behavior-generating mechanisms, it is conceivable that reference to behavior-generating mechanisms can answer ultimate Why questions. As I shall argue shortly in this paper, this is exactly what is at stake in the debate over whether or not reference to cultural selection can qualify as ultimate explanation.
